# Supplementary material for: Virus-mediated export of chromosomal DNA in plants
Source: Nat Commun. 2018 Dec 13;9:5308. doi: 10.1038/s41467-018-07775-w (PMC6293997; doi:10.1038/s41467-018-07775-w)
Supplement: Supplementary file 1 — Supplementary Information [file 41467_2018_7775_MOESM1_ESM.pdf]

Supplementary Information

Virus-mediated export of chromosomal DNA in plants

Catoni et al.

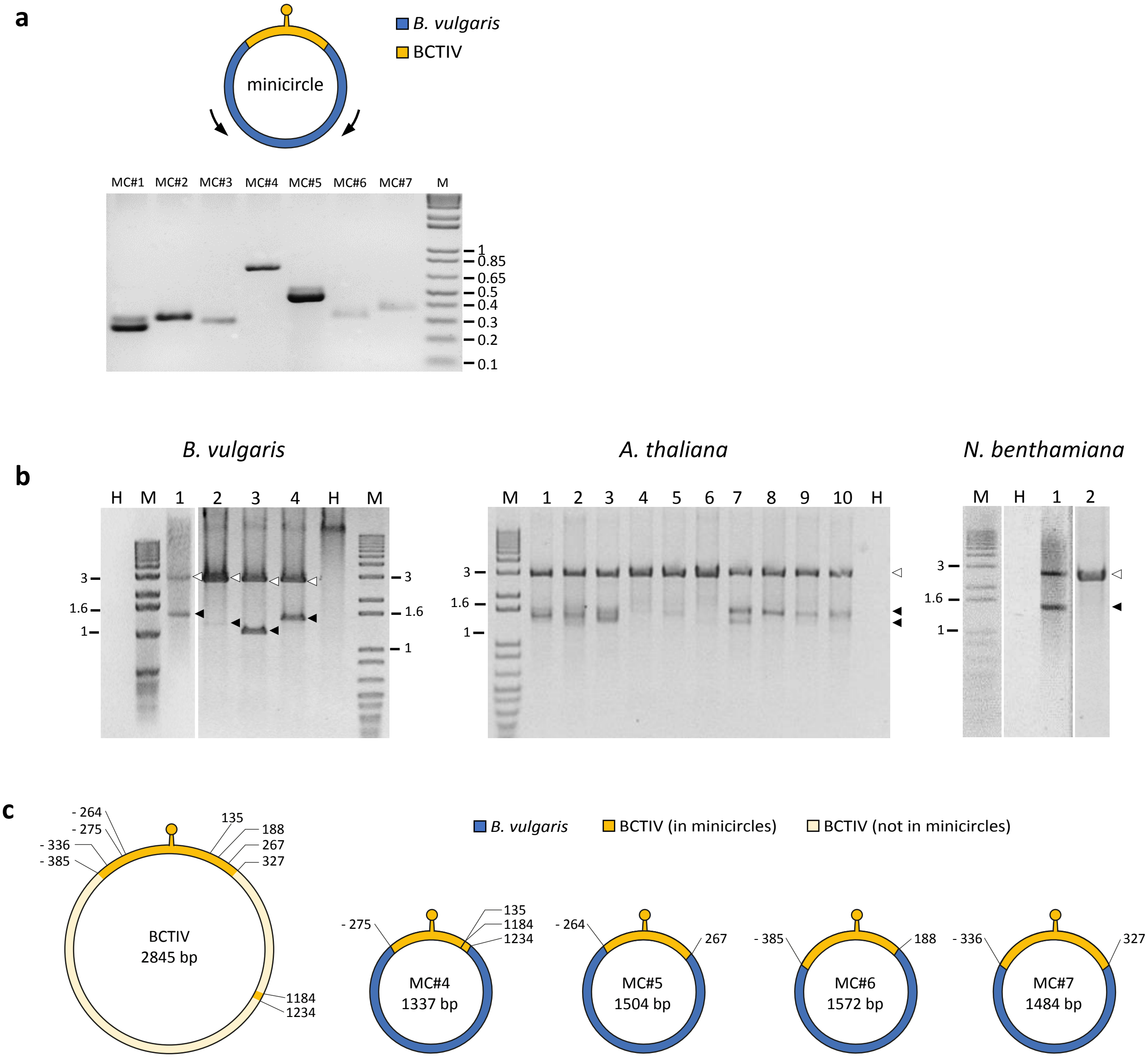

Supplementary Figure 1. Minicircles are circular hybrid DNA molecules detected in BCTIV-infected *B. vulgaris* plants.

a *In vivo* validation of the origin of the non-viral sequences of minicircles MC#1 to #7 performed by PCR on DNA from healthy *B. vulgaris*. The approximate positions of primers designed on the non-viral portion of minicircles (in blue) are indicated by arrows. Molecular markers (kb) are indicated. Source data are provided as a Source Data file.

b DNA fragments obtained after RCA and enzymatic digestion (see Methods) of plant samples (numbered above each lane) inoculated by BCTIV under controlled conditions and collected at 4 wpi. H, healthy control plants. The putative full-length BCTIV genome and associated smaller molecules are indicated by white and black arrows, respectively. M, molecular markers with sizes (kb) indicated. Source data are provided as a Source Data file.

c Scheme of the minicircles recovered from *B. vulgaris* plants shown in **b**; MC#4 originates from plant 1, MC#5 from plant 4, and MC#6 and #7 from plant 2. Color code and the coordinates are as in Figure 1b. The complete sequences of the minicircles are reported in Supplementary Data 2.

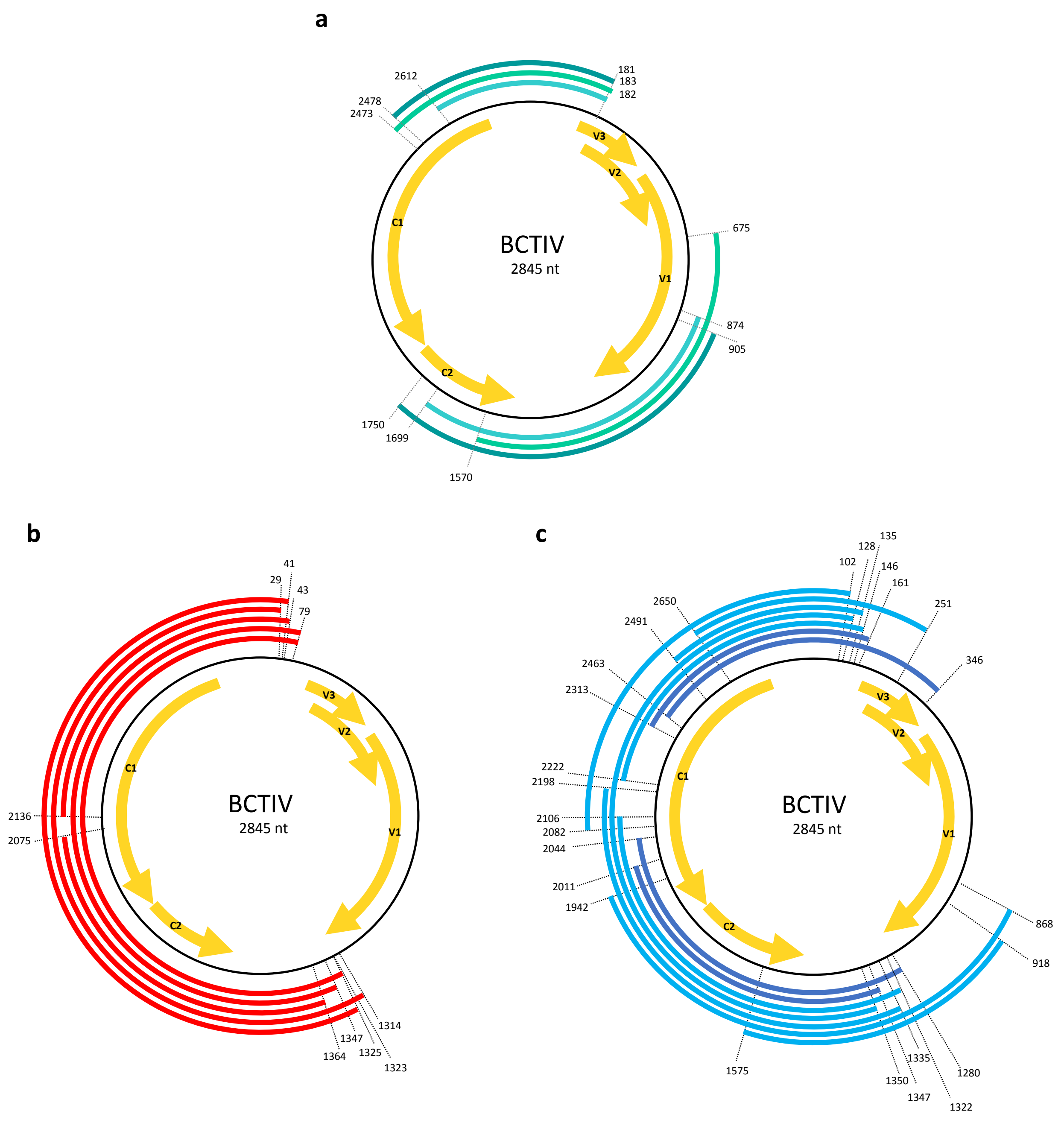

Supplementary Figure 2. Schematic representation of BCTIV defective molecules cloned from BCTIV-infected plants.

Defective DNAs cloned from *B. vulgaris* (**a**), *N. benthamiana* (**b**), and *A. thaliana* (**c**) displayed relative to the circular genome of BCTIV. The defective molecules were derived from three *B. vulgaris* plants (plants Nos. 2, 3, and 4 of Supplementary Fig. 1b; lines with three shades of green), one *N. benthamiana* plant (plant No. 1 of Supplementary Fig. 1b; red lines), and two *A. thaliana* plants (plants Nos. 7 and 8 of Supplementary Fig. 1b; lines with two shades of blue). Defective viral derivatives were cloned from RCA products digested with *Apa*I (**a** and **c**) or *Eco*RI (**b**).

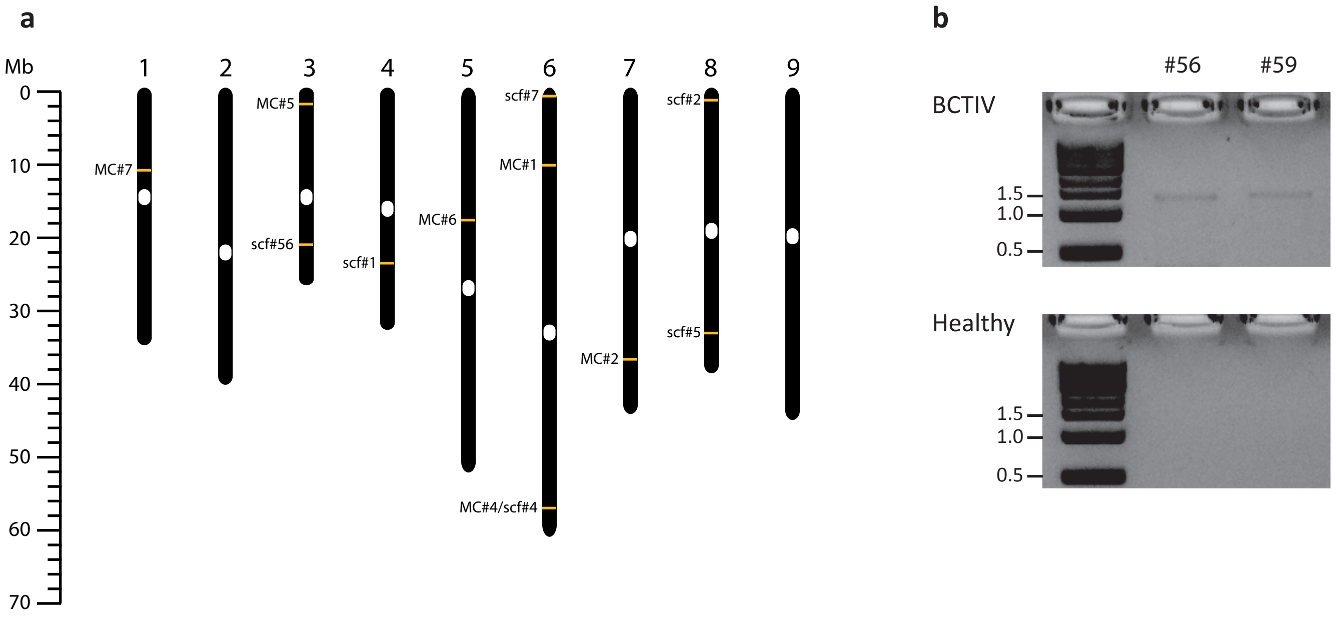

Supplementary Figure 3. Minicircles include DNA derived from *B. vulgaris* chromosomes.

a Schematic representation (yellow bars) of the chromosomal positions of the non-viral sequences present in eleven minicircles. The nine *B. vulgaris* chromosomes are drawn to the scale (Mb) shown on the left (sequences from RefBeet-1.2.2). Approximate positions of centromeres are indicated by white dots.

b *In vivo* validation by inverse PCR of the circular nature of scaffolds #56 and #59. The amplified fragments were sequenced and associated to a single minicircle (sequence reported in Supplementary Data 1). DNA from mock-inoculated *B. vulgaris* (Healthy) was used as a negative control. Molecular markers (kb) are shown on the left. Source data are provided as a Source Data file.

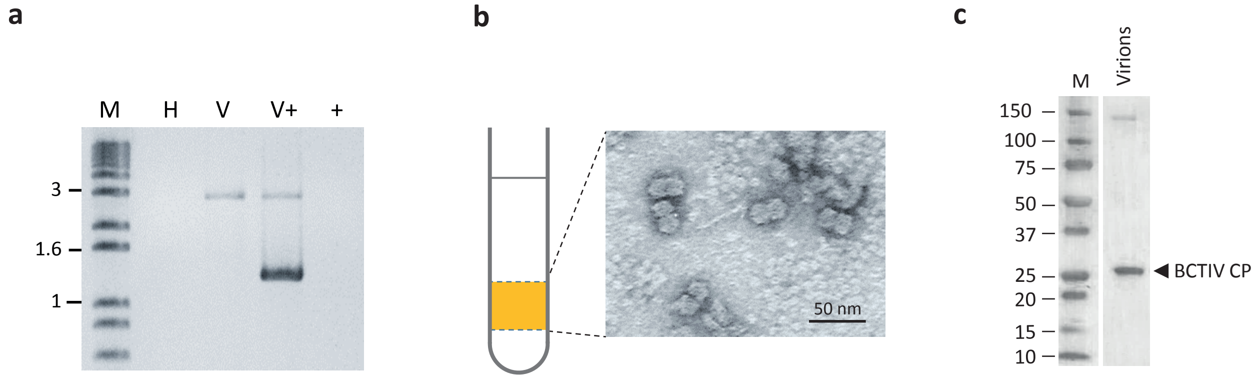

Supplementary Figure 4. Minicircle DNA replicates in plants and is encapsidated in BCTIV virions.

a PCR amplification of viral DNA from beet plants inoculated with BCTIV alone (V), with BCTIV and MC#1 (V+), or with MC#1 alone (+). H, healthy control. M, molecular markers (kb). Source data are provided as a Source Data file.

b Electron micrograph of geminated BCTIV virions purified by sucrose gradient from *N. benthamiana* plants inoculated with BCTIV and MC#1, collected at 4 wpi. Source data are provided as a Source Data file.

c SDS-PAGE of proteins extracted from BCTIV virions, stained with Coomassie blue. An arrow indicates the band of 28 kDa, the expected size of the BCTIV coat protein (CP). Lane M, molecular markers with sizes shown on the left (kDa). Source data are provided as a Source Data file.

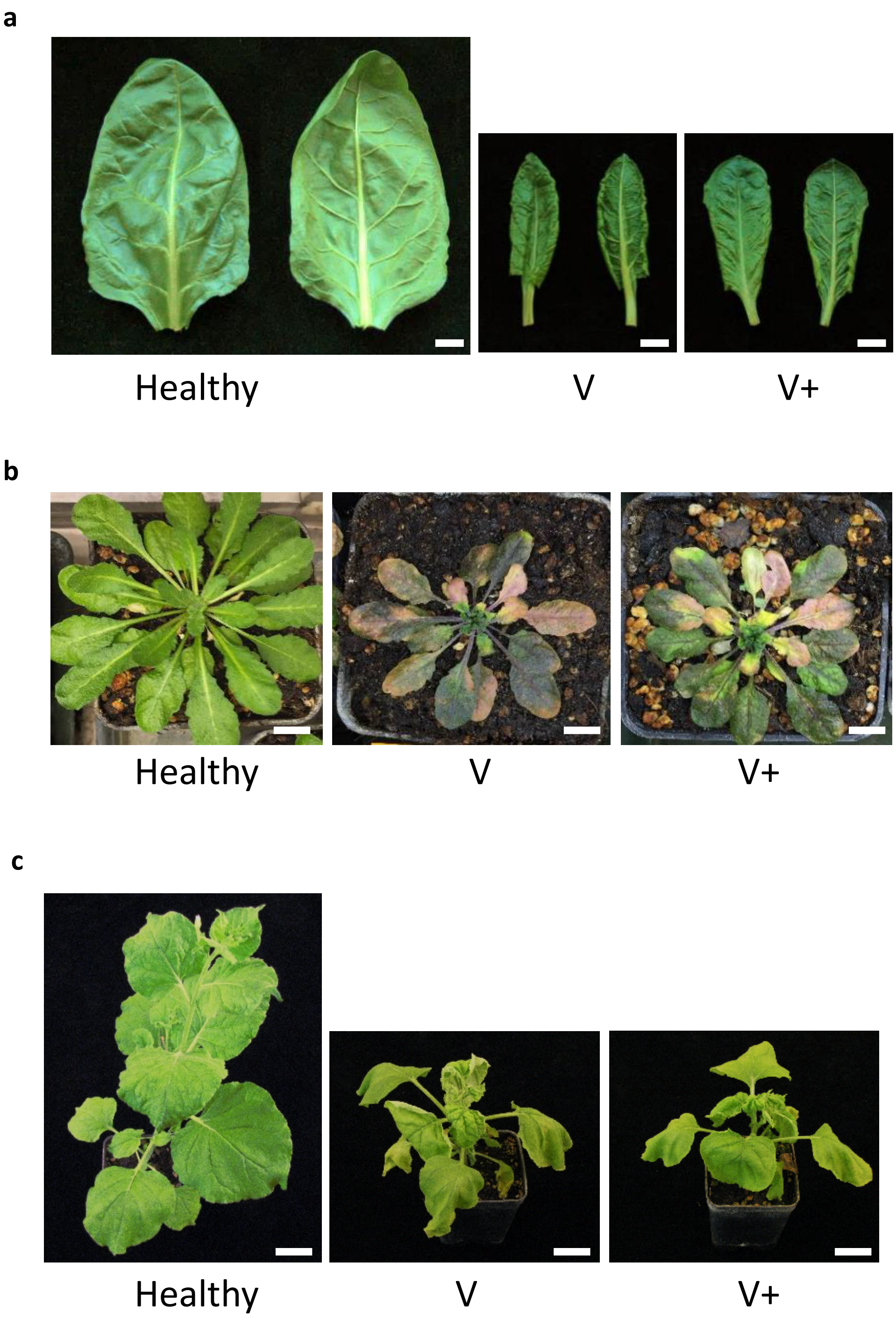

Supplementary Figure 5. Images of leaves and plants inoculated with BCTIV (V) or with BCTIV and MC#1 (V+), or mock-inoculated plants used as controls (Healthy).
a, *B. vulgaris* (white bars 1 cm), **b**, *A. thaliana*, (white bars 1 cm), and **c**, *N. benthamiana* (white bars 3 cm). Plants were photographed at 3 wpi.

Supplementary Table 1. Features of the defective molecules cloned following RCA from plants artificially infected by BCTIV. Identity with BCTIV-Siv (JX082259) is over 99% for all molecules.

Plant of origin	Clone	Length (nt)	BCTIV-Siv Coverage (%)
B. vulgaris[(a)]	DEF-Bv7/3	1378	100
	DEF-Bv8/9	1238	100
	DEF-Bv9/7	1428	100
N. benthamiana[(b)]	DEF-Nb1/14	1561	100
	DEF-Nb1/12	1595	100
	DEF-Nb1/11	1573	100
	DEF-Nb1/7	1451	100
	DEF-Nb1/6	1556	100
A. thaliana[(c)]	DEF-At1/2	1492	100
	DEF-At1/7	1359	100
	DEF-At2/1	1541	100
	DEF-At2/3	1620	100
	DEF-At2/4	1346	100
	DEF-At2/7	1546	98[(d)]
	DEF-At2/8	1521	100

(a) *B. vulgaris* clones derive from three plants (Bv7, Bv8 and Bv9)

(b) *N. benthamiana* clones derive from a single plant (Nb1)

(c) *A. thaliana* clones derive from two plants (At1 and At2)

(d) Presence of an insertion of unknown origin AATTATACTAGATATTTATAAGGACAG (27bp)

Supplementary Table 2. Primers used in this study

Name	***Sequence 5' > 3'***
#MC1 amplification	
MC#1_173F	GACGAGTCCGACACAATACG
MC#1_893R	TGCGAGATGGTAGTGGTGAA
1L-5_342F	CGTCCTTTGTTTTCATTCTGC
1L-5_597R	TGAAGACTCGAATGCACTTGT
#MC2 amplification	
1BC_I_664F	AGGCTAGTGTTAGATCTTTTGCA
1BC_1_552F	CCACATTCCCAAACATACCA
1BC_1_979R	CACCTTGATGACGACGTGAC
#MC3 amplification	
1-8BC-734F	CATCCAAGATTCAAGCCCTTT
1-8BC-1011R	TTTGCAGGTGTATCAACAGGTC
#MC4 amplification	
6ast11nv_308F	TGAACTGAAATTAAGTCTAAAAGAACA
6ast11nv_1010R	TTAGTAACAACGATAAAAATTTGGTG
#MC5 amplification	
MCBv9-1F	CCCTGCCAAATCTAGACCCA
MCBv9-1R	ACGAGTTGGGGAAGTAAAAGAAC
#MC6 amplification	
BV7_9_857F	TTTGTGTTTGCGTCCCCAAA
MCBv7-9F	CACTGAGTTTCCCACAAGAGTC
MCBv7-9R	GGTGTCAAAGATTTCAAGGTTCG
#MC7 amplification	
MCBv7-18F	TGTACTCCCTCCATTCCAGA
MCBv7-18R	AGTGTGGGCCAAAAGAGTTT
Inverse PCR for validation of scaffolds circular nature	
mcirc_beta_scf1_F	TCCAATACCGTCGTTTTGGTT
mcirc_beta_scf1_R	TGTGGGATCTTGTTAGATTCGTCT
mcirc_beta_scf2_F	CGCGCACAAAATAGGATGCA
mcirc_beta_scf2_R	TCTGAAATCTTGACACATTCACAAC
mcirc_beta_scf4_F	AGCTAAATTAAATTCACTCCACTACAACT
mcirc_beta_scf4_R	AGAAATTTATGCAGATAAATACGTCAGACT
mcirc_beta_scf5_F	AGAAAACACAGAAACGGCCA
mcirc_beta_scf5_R	TGTCCGTTGTAGTACGAGATTCA
mcirc_beta_scf7_F	GGCTTAACCTTTGCGGCTTT
mcirc_beta_scf7_R	ACAATTGCGACACCAGTACAA
mcirc_beta_scf33_F	TGTTCCAAAAACATGCTCAGCA
mcirc_beta_scf33_R	TGTCAATGCACAATTTTGCCCA
mcirc_beta_scf56_F	TGTGGAACATCTCCCGCTTT
mcirc_beta_scf56_R	TGACGATCGTAGTTTTTATGAAGGA

mcirc_beta_scf59_F	TGTCGGACCAAACTTGTGCT	
mcirc_beta_scf59_R	TATGGAGTGTGCCTAGGCCT	
MC#1 transcription		
MC#1_176F	GAGTCCGACACAATACGTTCTC	
MC#1_406R	ACACTTGTGCCATTGTGCAT	
BCTIV amplification		
BCTIV_42F	ACGGTTGAGTGGGGAACAC	
BCTIV_2820R	TCCCTCTTCCTCCCTCTTCA	
BCTIV_75F	TTAAAGTAAAGTAGCACTAAGTGGG	
BCTIV_2744R	ATTGTACGGAAGAGGGAAAC	
qPCR on total DNA		
qPCR_scaf1_betaBV_F	CATGTATTCTCCCCTCGTCACA	
qPCR_scaf1_betaBV_R	TGGGTTGGATTACTGGAACACA	
qPCR_scaf2_betaBV_F	ACGCGCACAAAATAGGATGC	
qPCR_scaf2_betaBV_R	ACGGGATAGATGTAGCATTGTCC	
qPCR_scaf4_betaBV_F	ACGTTCTAATGCAACGGTTGAC	
qPCR_scaf4_betaBV_R	TGCATTAGTGTTATCCGTTGCAAA	
qPCR_scaf5_betaBV_F	TGTTGACGCACAATTTTGACCA	
qPCR_scaf5_betaBV_R	GGATAGTGACCTAGCACACAACA	
qPCR_scaf7_betaBV_F	GTTCGCAATTAACAATTCACCAACTT	
qPCR_scaf7_betaBV_R	TGTAACAGCTCGTCTTATCCATTGA	
qPCR_scaf33_betaBV_F	TGGGCAAAATTGTGCATTGACA	
qPCR_scaf33_betaBV_R	AGCCCATATTATCCCAAGTTCCC	
qPCR_scaf56_betaBV_F	ATTTCCTTCATAAAAACTACGATCGTCA	
qPCR_scaf56_betaBV_R	TCCCTATAAAAGCGGGAGATGTTC	
BvGAPDH_F	TGCACCGATGTTTGTTGTCG	
BvGAPDH_R	GGGAGCAAGGCAATTTGTGG	
qPCR_BCTIV_C1_F	TTTCCTGTTCTGGATGGTCCG	
qPCR_BCTIV_C1_R	CTCCAGGAGCCAGACAACAG	
Probe synthesis		
MC#1_682F	TGATCACCCATGTTAATTCAGC	
MC#1_925R	ACGTCAATGGGAACCTGTATTC	
BCTIRV-F	TACAAGTATGGCGGTTC	(Soleimani et al., 2013)
BCTIRV-R	GAGTAAAGCATTCTCCTTCAC	(Soleimani et al., 2013)

Supplementary Table 3. Metrics from analysis of genome wide DNA sequencing

Plant	Condition	name	Total paired	Trimming (trimmomatic)		Mapping (Segemehl)			Total kept reads	Genome coverage (average)
				Final trim	% kept reads	Mapping efficiency	Multimatches	Duplicated		
B. vulgaris	Healthy	B vulgaris HH	15,901,042	15,109,660	95.02%	91.49%	40.63%	3.03%	13,823,828	3.57
B. vulgaris	BCTIV-infected	B vulgaris BT	30,110,885	29,073,207	96.55%	66.31%	28.87%	3.57%	19,278,444	4.92
A. thaliana	BCTIV-infected	Col-0 BT_7	8,916,726	8,606,893	96.53%	90.15%	22.40%	3.39%	7,759,114	9.50
A. thaliana	BCTIV-infected	Col-0 BT_8	14,180,521	13,429,965	94.71%	98.77%	25.22%	7.19%	13,264,776	15.18
