# Supplementary material for: Virus-mediated export of chromosomal DNA in plants
Source: Nat Commun. 2018 Dec 13;9:5308. doi: 10.1038/s41467-018-07775-w (PMC6293997; doi:10.1038/s41467-018-07775-w)
Supplement: Supplementary file 3 — Description of Additional Supplementary Files [file 41467_2018_7775_MOESM3_ESM.docx]

**Description of Additional Supplementary Files**

**File Name:** Supplementary Data 1

**Description:** Sequences of fragments amplified with inverse PCR from a single BCTIV infected plant.

**File Name:** Supplementary Data 2

**Description:** Sequences of cloned minicircles.

**File Name:** Supplementary Data 3

**Description:** Sequence of the filtered scaffolds obtained from the NGS analysis.

**File Name:** Supplementary Data 4

**Description:** List of BLAST hits of scaffold sequences on B. vulgaris genome.

**File Name:** Supplementary Data 5

**Description:** Source data underlying Figs 1a, 2c, f, 3a-e, and Supplementary Figs 1a, b, 3b, and 4a-c
