# Supplementary material for: Virus-mediated export of chromosomal DNA in plants
Source: Nat Commun. 2018 Dec 13;9:5308. doi: 10.1038/s41467-018-07775-w (PMC6293997; doi:10.1038/s41467-018-07775-w)
Supplement: Supplementary file 4 — Supplementary Data 1 [file 41467_2018_7775_MOESM4_ESM.docx]

**Supplementary Data 1**

Sequences of fragments amplified with inverse PCR from a single BCTIV-infected plant. Primers used for amplification are reported in Supplementary Table 2 and were designed on the *B. vulgaris* genomic sequence of the eight scaffolds assembled in the DNAseq analysis. Two clones have been sequenced per fragment. Sequence from the pGEM-T cloning vector has been removed.

Legend:

Yellow = BCTIV genome

Underlined = stem loop structure

**BOLD** = start codon (ATG) of viral ORFs

Blue = *B. vulgaris* genome

Dark Green = forward primers used in the inverse PCR reaction

Light Green = reverse primers used in the inverse PCR reaction

>beta_scf1_1

TGTGGGATCTTGTTAGATTCGTCTGAATGTATATTTTCCGAATATGTATATATATATATATATATATATACACATATGATTCTAATAAGGTCTTCAGTTTCTCGAATATATAATCTTTAAAATCCCCCCCTATTTGAGAATATGTTAGAAAAGCATTTTTTTTTTGAAAACGTAAATAACCGGGTTGGTTAGGGGTAGGTCTGTTTCTGTTTGGAACACGTGG**CAT**GTTTGTTTTTGTTTCCCTCTTCCGTACAATGAATGAAAAGAAATTGTATATATGAGAGGAAGAGGAACTTAAGAGGAATGAGGAACTGAAGAGGGAGGAAGAGGGATAAATAGGTAAACGCGGATAAGATTCCCCGCGTTTACCTTTCCACGTGGCGTATGCTGAGTGGGCGGACGGTTGAGTGGGGAACACGCTTTACTTTAATTTAAAGTAAAGTAGCACTAAGTGGGCCCCACATTTTGAATGGATATATTTCTATTTTAGTTTTGATTTTGAATCATACAATAGTAATGCTTATAGTATATTTCTATGTATTTAACATGATATCTACTTTTTCATTTGAAACCAAAACGACGGTATTGGA

>beta_scf1_2

TGTGGGATCTTGTTAGATTCGTCTGAATGTATATTTTCCGAATATATATATATATATATATATATACACATATGATTCTAATAAGGTCTTCAGTTTCTCGAATATATAATCTTTAAAATCCCCCCCTATTTGAGAATATGTTAGAAAAGCATTTTTTTTTTGAAAACGTAAATAACCGGGTTGGTTAGGGGTAGGTCTGTTTCTGTTTGGAACACGTGG**CAT**GTTTGTTTTTGTTTCCCTCTTCCGTACAATGAATGAAAAGAAATTGTATATATGAGAGGAAGAGGAACTTAAGAGGAATGAGGAACTGAAGAGGGAGGAAGAGGGATAAATAGGTAAACGCGGATAAGATTCCCCGCGTTTACCTTTCCACGTGGCGTATGCTGAGTGGGCGGACGGTTGAGTGGGGAACACGCTTTACTTTAATTTAAAGTAAAGTAGCACTAAGTGGGCCCCACATTTTGAATGGATATATTTCTATTTTAGTTTTGATTTTGAATCATACAATAGTAATGCTTATAGTATATTTCTATGTATTTAACATGATATCTACTTTTTCATTTGAAACCAAAACGACGGTATTGGA

>beta_ scf2_1

CGCGCACAAAATAGGATGCAAATACCAAAAGGAAATCTATATAAATGGACAATGCTACATCTATCCCGTTAATAATTAATCTACTAAAAATAAAAAATTAGAAAACTATTAAAATTAATAAATTATACTCCCTCTGTTTTTTTTTATCTTTCTCACTTAGAATCTTGACACTATTCATGTATAGAGAGAATGTTTCTGTTTGGAACACGTGG**CAT**GTTTGTTTTTGTTTCCCTCTTCCGTACAATGAATGAAAAGAAATTGTATATATGAGAGGAAGAGGAACTTAAGAGGAATGAGGAACTGAAGAGGGAGGAAGAGGGATAAATAGGTAAACGCGGATAAGATTCCCCGCGTTTACCTTTCCACGTGGCGTATGCTGAGTGGGCGGACGGTTGAGTGGGGAACACGCTTTACTTTAATTTAAAGTAAAGTAGCACTAAGTGGGCCCCACATTTTATTTCTTTAAAGACTTACTTTTTAAGTCTTCTAAATGCGTATATTATACGTATAA**ATG**ATGGTCTGTCCCCCTAGTTGTGAATGTGTCAAGATTTCAGA

>beta_ scf2_2

CGCGCACAAAATAGGATGCAAATACCAAAAGGAAATCTATATAAATGGACAATGCTACATCTATCCCGTTAATAATTAATCTACTAAAAATAAAAAATTAGAAAACTATTAAAATTAATAAATTATACTCCCTCTGTTTTTTTTTATCTTTCTCACTTAGAATCTTGACACTATTCATGTATAGAGAGAATGTTTCTGTTTGGAACACGTGG**CAT**GTTTGTTTTTGTTTCCCTCTTCCGTACAATGAATGAAAAGAAATTGTATATATGAGAGGAAGAGGAACTTAAGAGGAATGAGGAACTGAAGAGGGAGGAAGAGGGATAAATAGGTAAACGCGGATAAGATTCCCCGCGTTTACCTTTCCACGTGGCGTATGCTGAGTGGGCGGACGGTTGAGTGGGGAACACGCTTTACTTTAATTTAAAGTAAAGTAGCACTAAGTGGGCCCCACATTTTATTTCTTTAAAGACTTACTTTTTAAGTCTTCTAAATGCGTATATTATACGTATAA**ATG**ATGGTCTGTCCCCCTAGTTGTGAATGTGTCAAGATTTCAGA

>beta_ scf4_1

AGAAATTTATGCAGATAAATACGTCAGACTAATAAGGTCTTCAGTTTCTCGAATATATAATCTTTAAAATCCCCCCCTATTTGAGAATATGTTAGAAAAGCATTTTTTTTTGAAAACGTAAATAACCGGGTTGGTTAGGGGTAGGTCTGTTTCTGTTTGGAACACGTGG**CAT**GTTTGTTTTTGTTTCCCTCTTCCGTACAATGAATGAAAAGAAATTGTATATATGAGAGGAAGAGGAACTTAAGAGGAATGAGGAACTGAAGAGGGAGGAAGAGGGATAAATAGGTAAACGCGGATAAGATTCCCCGCGTTTACCTTTCCACGTGGCGTATGCTGAGTGGGCGGACGGTTGAGTGGGGAACACGCTTTACTTTAATTTAAAGTAAAGTAGCACTAAGTGGGCCCCACATTTTATTTCTTTAAAGACTTACTTTTTAATACAAATATGTATGCTACTTTGTTTGGTAATTGTAGGGCTTATTTCTACTAATAGTAACAAAATTGGAAATAAATTGAATTAAATTTAATTTAGTTGTAGTGGAGTGAA

>beta_ scf4_2

AGAAATTTATGCAGATAAATACGTCAGACTAATAAGGTCTTCAGTTTCTCGAATATATAATCTTTAAAATCCCCCCCTATTTGAGAATATGTTAGAAAAGCATTTTTTTTTGAAAACGTAAATAACCGGGTTGGTTAGGGGTAGGTCTGTTTCTGTTTGGAACACGTGG**CAT**GTTTGTTTTTGTTTCCCTCTTCCGTACAATGAATGAAAAGAAATTGTATATATGAGAGGAAGAGGAACTTAAGAGGAATGAGGAACTGAAGAGGGAGGAAGAGGGATAAATAGGTAAACGCGGATAAGATTCCCCGCGTTTACCTTTCCACGTGGCGTATGCTGAGTGGGCGGACGGTTGAGTGGGGAACACGCTTTACTTTAATTTAAAGTAAAGTAGCACTAAGTGGGCCCCACATTTTATTTCTTTAAAGACTTACTTTTTAATACAAATATGTATGCTACTTTGTTTGGTAATTGTAGGGCTTATTTCTACTAATAGTAACAAAATTGGAAATAAATTGAATTAAATTTAATTTAGTTGTAGTGGAGTGAATTTAATTTAGCT

>beta_ scf5_1

TGTCCGTTGTAGTACGAGATTCAAAGGAAAATTCTAGGGCTCGTGTCCTCCTTGGTGATGTACTCGAATGCTCGCTTTGGGGCGTTGAGTCCGTCGATTCTTGGGTGAATCGTTCGACCGTCTGGGAGTATGATGTTGAAAAACAGGTTCCCATTGACGTCGAGTTTCTTGTCACACTGGATAATACAGTGTGTGTGGAATCCTCCTTCAGGTTGTTCTTCAGTAGGCTGATGATGTTCTAGTGATACTGCAAGGAATAGAATTACATATGATTCTAATAAGGTCTTCAGTTTCTCGAATATATAATCTTTAAAATCCCCCCCTATTTGAGAATATGTTAGAAAAGCATTTTTTTTTTGAAAACGTAAATAACCGGGTTGGTTAGGGGTAGGTCTGTTTCTGTTTGGAACACGTGG**CAT**GTTTGTTTTTGTTTCCCTCTTCCGTACAATGAATGAAAAGAAATTGTATATATGAGAGGAAGAGGAACTTAAGAGGAATGAGGAACTGAAGAGGGAGGAAGAGGGATAAATAGGTAAACGCGGATAAGATTCCCCGCGTTTACCTTTCCACGTGGCGTATGCTGAGTGGGCGGACGGTTGAGTGGGGAACACGCTTTACTTTAATTTAAAGTAAAGTAGCACTAAGTGGGCCCCACATTTTATTTCTTTAAAGACTTACTTTTTAAGTCTTCTAAATGCGTATATTATACGTATAA**ATG**ATGGTCTGTATTCCCGACTGGTTATTTCCGCGCTTTTTGGCCGTTTCTGTGTTTTCT

>beta_ scf5_2

TGTCCGTTGTAGTACGAGATTCAAAGGAAAATTCTAGGGCTCGTGTCCTCCTTGGTGATGTACTCGAATGCTCGCTTTGGGGCGTTGAGTCCGTCGATTCTTGGGTGAATCGTTCGACCGTCTGGGAGTATGATGTTGAAAAACAGGTTCCCATTGACGTCGAGTTTCTTGTCACACTGGATAATACAGTGTGTGTGGAATCCTCCTTCAGGTTGTTCTTCAGTAGGCTGATGATGTTCTAGTGATACTGCAAGGAATAGAATTACATATGATTCTAATAAGGTCTTCAGTTTCTCGAATATATAATCTTTAGAATCCCCCCCTATTTGAGAATATGTTAGAAAAGCATTTTTTTTTTGAAAACGTAAATAACCGGGTTGGTTAGGGGTAGGTCTGTTTCTGTTTGGAACACGTGG**CAT**GTTTGTTTTTGTTTCCCTCTTCCGTACAATGAATGAAAAGAAATTGTATATATGAGAGGAAGAGGAACTTAAGAGGAATGAGGAACTGAAGAGGGAGGAAGAGGGATAAATAGGTAAACGCGGATAAGATTCCCCGCGTTTACCTTTCCACGTGGCGTATGCTGAGTGGGCGGACGGTTGAGTGGGGAACACGCTTTACTTTAATTTAAAGTAAAGTAGCACTAAGTGGGCCCCACATTTTATTTCTTTAAAGACTTACTTTTTAAGTCTTCTAAATGCGTATATTATACGTATAA**ATG**ATGGTCTGTATTCCCGACTGGTTATTTCCGCGCTTTTTGGCCGTTTCTGTGTTTTCT

>beta_ scf7_1

ACAATTGCGACACCAGTACAAAGTACAAAGAAGGGGAGAATTACATATGATTCTAATAAGGTCTTCAGTTTCTCGAATATATAATCTTTAAAATCCCCCCCTATTTGAGAATATGTTAGAAAAGCATTTTTTTTTTGAAAACGTAAATAACCGGGTTGGTTAGGGGTAGGTCTGTTTCTGTTTGGAACACGTGG**CAT**GTTTGTTTTTGTTTCCCTCTTCCGTACAATGAATGAAAAGAAATTGTATATATGAGAGGAAGAGGAACTTAAGAGGAATGAGGAACTGAAGAGGGAGGAAGAGGGATAAATAGGTAAACGCGGATAAGATTCCCCGCGTTTACCTTTCCACGTGGCGTATGCTGAGTGGGCGGACGGTTGAGTGGGGAACACGCTTTACTTTAATTTAAAGTAAAGTAGCACTAAGTGGGCCCCACATTTTATTTCTTTAAAGACTTACTTTTTAAGTCTTCTAAATGCGTATATTATACGTATAA**ATG**ATGGTCTGTATTCCCGACTGGTTATTTCTACTCTTCGTATTAGTACGATTCTTCAGTCGGGAATTAATTTCT**ATG**GTACCTTTCAGAGTGAGCGAATTTCCGCGAAGCTATCCAGCCTTGCTAGCCGTTTCGACGAGCTGTTTCTTGCGTTACAACAAGTCCCGAAAAGCCGCAAAGGTTAAGCC

>beta_ scf7_2

ACAATTGCGACACCAGTACAAAGTACAAAGAAGGGGAGAATTACATATGATTCTAATAAGGTCTTCAGTTTCTCGAATATATAATCTTTAAAATCCCCCCCTATTTGAGAATATGTTAGAAAAGCATTTTTTTTTTGAAAACGTAAATAACCGGGTTGGTTAGGGGTAGGTCTGTTTCTGTTTGGAACACGTGG**CAT**GTTTGTTTTTGTTTCCCTCTTCCGTACAATGAATGAAAAGAAATTGTATATATGAGAGGAAGAGGAACTTAAGAGGAATGAGGAACTGAAGAGGGAGGAAGAGGGATAAATAGGTAAACGCGGATAAGATTCCCCGCGTTTACCTTTCCACGTGGCGTATGCTGAGTGGGCGGACGGTTGAGTGGGGAACACGCTTTACTTTAATTTAAAGTAAAGTAGCACTAAGTGGGCCCCACATTTTATTTCTTTAAAGACTTACTTTTTAAGTCTTCTAAATGCGTATATTATACGTATAA**ATG**ATGGTCTGTATTCCCGACTGGTTATTTCTACTCTTCGTATTTAGTACGATTCTTCAGTCGGGAATTAATTTCT**ATG**GTACCTTTCAGAGTGAGCGAATTTCCGCGAAGCTATCCAGCCTTGCTAGCCGTTTCGACGAGCTGTTTCTTGCGTTACAACAAGTCCCGAAAAGCCGCAAAGGTTAAGCC

>beta_ scf33_1A

TGTCAATGCACAATTTTGCCCATAAATAACTTTAGTTATGTACTTGTATTTGTAAAAATTATGCAAATGAAATACTAAAAAATTACACACTGAGACGTATTAAATAAGATCTCATATGACCATATTTATGTGTGATTGATCATCATATACTGTTCAAGTCTGTTGGGCAGGACTCTCGTATGTTACTGAAGAACTCCTCCTTGGTGTTGGATGAATCAAGAATTCGTCTCCATTCCACGTTGCTGTTGCCAATACTGTTTGGAGAGCGCCCTCCCAGTCGAAGCTCTCCAAATGTTCTAGGGCTCGTGTCCTCCTTGGTGATGTACTCGAATGCTCGCTTTGGGGCGTTGAGTCCGTCGATTCTTGGGTGAATCGTTCGACCGTCTGGGAGTATGATGTTGAAAAACAGGTTCCCATTGACGTCGAGTTTCTTGTCACACTGGATAATACAGTGTGTGTGGAATCCTCCTTCAGGTTGTTCTTCAGTAGGCTGATGATGTTCTAGTGATACTGCAAGGAATAGAATTACACATGATTCTAATAAGGTCTTCAGTTTCTCGAATATATAATCTTTAAAATCCCCCCCTATTTGAGAATATGTTAGAAAAGCATTTTTTTTTTGAAAACGTAAATAACCGGGTTGGTTAGGGGTAGGTCTGTTTCTGTTTGGAACACGTGG**CAT**GTTTGTTTTTGTTTCCCTCTTCCGTACAATGAATGAAAAGAAATTGTATATATGAGAGGAAGAGGAACTTAAGAGGAATGAGGAACTGAAGAGGGAGGAAGAGGGATAAATAGGTAAACGCGGATAAGATTCCCCGCGTTTACCTTTCCACGTGGCGTATGCTGAGTGGGCGGACGGTTGAGTGGGGAACACGCTTTACTTTAATTTAAAGTAAAGTAGCACTAAGTGGGCCCCACATTATGATGTGAAGTATATAATGATTTGATTTTATTAGTTCACAAATTCTGAAATTGAGTTTATATTTTTGTTATTGTCAC

>beta_ scf33_2A

CCCAGTCGAAGCTCTCCAAATGTTCTAGGGCTCGTGTCCTCCTTGGTGATGTACTCGAATGCTCGCTTTGGGGCGTTGAGTCCGTCGATTCTTGGGTGAATCGTTCGACCGTCTGGGAGTATGATGTTGAAAAACAGGTTCCCATTGACGTCGAGTTTCTTGTCACACTGGATAATACAGTGTGTGTGGAATCCTCCTTCAGGTTGTTCTTCAGTAGGCTGATGATGTTCTAGTGATACTGCAAGGAATAGAATTACATATGATTCTAATAAGGTCTTCAGTTTCTCGAATATATAATCTTTAAGATCCCCCCCTATTTGAGAATATGTTAGAAAAGCATTTTTTTTTTGAAAACGTAAATAACCGGGTTGGTTAGGGGTAGGTCTGTTTCTGTTTGGAACACGTGG**CAT**GTTTGTTTTTGTTTCCCTCTTCCGTACAATGAATGAAAAGAAATTGTATATATGAGAGGAAGAGGAACTTAAGAGGAATGAGGAACTGAAGAGGGAGGAAGAGGGATAAATAGGTAAACGCGGATAAGATTCCCCGCGTTTACCTTTCCACGTGGCGTATGCTGAGTGGGCGGACGGTTGAGTGGGGAACACGCTTTACTTTAATTTAAAGTAAAGTAGCACTAAGTGGGCCCCACATTATGATGTGAAGTATATAATGATTTGATTTTATTAGTTCACAAATTCTGAAATTGAGTTTATATTTTTGTTATTGTCACCATAGTATTACCTTTTCAGAGAGTTACGAAATATTATTGTGAGAGAAAAAGTAGATATAAGAATAACAATTAAAAAAAAAATCAGGCGGAAGAATGTTAAATCCTACAAAAATTTTAGTCATAGATCCCTTAAAAAATAACATAGTCACATAGATGCTGAGCATGTTTTTGGAACA

>beta_ scf56_1

ATGACTTCTAAAATATACTACTTAATAATAAAGTTAAAAATACTCTCGCGCATTATACATGGTTCAAAAGGCTAGTATAATTATAAATATATATAGATTATATGCGTGTTAAAATTGTACATAGGAATGCTTAAGATTAGAGTTGTATATGGCCCGATCTAGCACAAGTTTGGTCCGACATGTCATAACCGAGACTAGCTGAACACGGCAAGTTGGGTTGTGACATATGGAGTGTGCCTAGGCCTCCTTTTTTTAAAATTCAGCATAATCATCCTCCTTCAGGTTGTTCTTCAGTAGGCTGATGATGTTCTAGTGATACTGCAAGGAATAGAATTACATATGATTCTAATAAGGTCTTCAGTTTCTCGAATATATAATCTTTAAAATCCCCCCCTATTTGAGAATATGTTAGAAAAGCATTTTTTTTTTGAAAACGTAGATAACCGGGTTGGTTAGGGGTAGGTCTGTTTCTGTTTGGAACACGTGG**CAT**GTTTGTTTTTGTTTCCCTCTTCCGTACAATGAATGAAAAGAAATTGTATATATGAGAGGAAGAGGAACTTAAGAGGAATGAGGAACTGAAGAGGGAGGAAGAGGGATAAATAGGTAAACGCGGATAAGATTCCCCGCGTTTACCTTTCCACGTGGCGTATGCTGAGTGGGCGGACGGTTGAGTGGGGAACACGCTTTACTTTAATTTAAAGTAAAGTAGCACTAAGTGGGCCCGGTGACTAGTTTAATCCATTTAGTGATTATGTCTAATTTTATACAAAAATTAGACCGTCTTTTTTTAATAATTTTTGAAATTACTAATATCGATGAATTTGTACTTTATCTAGTAATGTGGTCCTATAATGTTAACTTATTTTCAATCCCTATAAAAGCGGGAGATGTTCCACA

>beta_ scf56_2

ATGACTTCTAAAATATACTACTTAATAATAAAGTTAAAAATACTCTCGCGCATTATACATGGTTCAAAAGGCTAGTATAATTATAAATATATATAGATTATATGCGTGTTAAAATTGTACATAGGAATGCTTAAGATTAGAGTTGTATATGGCCCGATCTAGCACAAGTTTGGTCCGACATGTCATAACCGAGACTAGCTGAACACGGCAAGTTGGGTTGTGACATATGGAGTGTGCCTAGGCCTCCTTTTTTTAAAATTCAGCATAATCATCCTCCTTCAGGTTGTTCTTCAGTAGGCTGATGATGTTCTAGTGATACTGCAAGGAATAGAATTACATATGATTCTAATAAGGTCTTCAGTTTCTCGAATATATAATCTTTAAAATCCCCCCCTATTTGAGAATATGTTAGAAAAGCATTTTTTTTTTGAAAACGTAGATAACCGGGTTGGTTAGGGGTAGGTCTGTTTCTGTTTGGAACACGTGG**CAT**GTTTGTTTTTGTTTCCCTCTTCCGTACAATGAATGAAAAGAAATTGTATATATGAGAGGAAGAGGAACTTAAGAGGAATGAGGAACTGAAGAGGGAGGAAGAGGGATAAATAGGTAAACGCGGATAAGATTCCCCGCGTTTACCTTTCCACGTGGCGTATGCTGAGTGGGCGGACGGTTGAGTGGGGAACACGCTTTACTTTAATTTAAAGTAAAGTAGCACTAAGTGGGCCCGGTGACTAGTTTAATCCATTTAGTGATTATGTCTAATTTTATACAAAAATTAGACCGTCTTTTTTTAATAATTTTTGAAATTACTAATATCGATGAATTTGTACTTTATCTAGTAATGTGGTCCTATAATGTTAACTTATTTTCAATCCCTATAAAAGCGGGAGATGTTCCACA

>beta_ scf59_1

GAATGAAAAGAAATTGTATATATGAGAGGAAGAGGAACTTAAGAGGAATGAGGAACTGAAGAGGGAGGAAGAGGGATAAATAGGTAAACGCGGATAAGATTCCCCGCGTTTACCTTTCCACGTGGCGTATGCTGAGTGGGCGGACGGTTGAGTGGGGAACACGCTTTACTTTAATTTAAAGTAAAGTAGCACTAAGTGGGCCCGGTGACTAGTTTAATCCATTTAGTGATTATGTCTAATTTTATACAAAAATTAGACCGTCTTTTTTTTAATAATTTTTGAAATTACTAATATCGATGAATTTGTACTTTATCTAGTAATGTGGTCCTATAATGTTAACTTATTTTCAATCCCTATAAAAGCGGGAGATGTTCCACATTGCAAGATTTATTTGACGATCGTAGTTTTTATGAAGGAAATTTTTGTTTGATTCAACTTATGTATTTGTTTGATTCGATTGCTTATGTAGATGTCGTATGACTTTTTATTAATGAATTAATTTTGGCCCAGACTTTCATGTATACTTGGTTGAAGGATCAAGATGCTCCATAGTGACTAAGTTTCCATATATATTTAATGTGGAAGAGGATCCTTTAACGTTTAATGAGGCTATGAGGTCTCAAGATGCCGCTTTTTGGAAAGAGGCAATTGATGATGAGATGGATTCCATCATGGGAAATAACACTTGGATTCTAGCTGATTTACCTCCTAAGTGTAAACCAATTGGATGTAAATGGATTTTCAAAAAGAAAATGAAAGTAGATGGGACTATTGATAAGTTCAAAGCCCGTCTTGTAGCTCACATCTAAATTGATTTGGAAATTACATATGTAATTATTGTTCTTTAAGCACTCTTACTTCATGATTTTATAATTTTGTGTAACACGGGAAGGTAAGTGATTATTATTACATGACTTCTAAAATATACTACTTAATAATAAAGTTAAAAATACTCTCGCGCATTATACATGGTTCAAAAGGCTAGTATAATTATAAATATATATAGATTATATGCGTGTTAAAATTGTACATAGGAATGCTTAAGATTAGAGTTGTATATGGCCCGATCTAGCACAAGTTTGGTCCGACA

>beta_ scf59_2

TATGGAGTGTGCCTAGGCCTCCTTTTTTTAAAATTCAGCATAATCATCCTCCTTCAGGTTGTTCTTCAGTAGGCTGATGATGTTCTAGTGATACTGCAAGGAATAGAATTACATATGATTCTAATAAGGTCTTCAGTTTCTCGAATATATAATCTTTAAAATCCCCCCCTATTTGAGAATATGTTAGAAAAGCATTTTTTTTTTGAAAACGTAAATAACCGGGTTGGTTAGGGGTAGGTCTGTTTCTGTTTGGAACACGTGG**CAT**GTTTGTTTTTGTTTCCCTCTTCCGTACAATGAATGAAAAGAAATTGTATATATGAGAGGAAGAGGAACTTAAGAGGAATGAGGAACTGAAGAGGGAGGAAGAGGGATAAATAGGTAAACGCGGATAAGATTCCCCGCGTTTACCTTTCCACGTGGCGTATGCTGAGTGGGCGGACGGTTGAGTGGGGAACACGCTTTACTTTAATTTAAAGTAAAGTAGCACTAAGTGGGCCCGGTGACTAGTTTAATCCATTTAGTGATTATGTCTAATTTTATACAAAAATTAGACCGTCTTTTTTTTAATAATTTTTGAAATTACTAATATCGATGAATTTGTACTTTATCTAGTAATGTGGTCCTATAATGTTAACTTATTTTCAATCCCTATAAAAGCGGGAGATGTTCCACATTGCAAGATTTATTTGACGATCGTAGTTTTTATGAAGGAAATTTTTGTTTGATTCAACTTATGTATTTGTTTGATTCGATTGCTTATGTAGATGTCGTATGACTTTTTATTAATGAATTAATTTTGGCCCAGACTTTCATGTATACTTGGTTGAAGGATCAAGATGCTCCATAGTGACTAAGTTTCCATATATATTTAA
