# Supplementary material for: Virus-mediated export of chromosomal DNA in plants
Source: Nat Commun. 2018 Dec 13;9:5308. doi: 10.1038/s41467-018-07775-w (PMC6293997; doi:10.1038/s41467-018-07775-w)
Supplement: Supplementary file 5 — Supplementary Data 2 [file 41467_2018_7775_MOESM5_ESM.docx]

**Supplementary Data 2**

Fasta sequence of cloned minicircles from BCTIV-infected *Beta vulgaris* plants in field (MC#1, MC#2 and MC#3) and in experimental inoculation (MC#4, MC#5, MC#6 and MC#7). Minicircle sequences are reported starting from the centre of the stem loop structure, conventionally used as start of the BCTIV circular genome.

Legend:

Yellow = BCTIV genome

Underlined = stem loop structure

**BOLD** = start codon (ATG) of viral ORFs

Blue = *B. vulgaris* genome

Dark Green = forward primers used in the PCR validation on healthy *B. vulgaris*

Light Green = reverse primers used in the PCR validation on healthy *B. vulgaris*

>MC#1

CCCCGCGTTTACCTTTCCACGTGGCGTATGCTGAGTGGGCGGACGGTTGAGTGGGGAACACGCTTTACTTTAATTTAAAGTAAAGTAGCACTAAGTGGGCCCCACATTTTATTTCTTTAAAGACTTACTTTTTAAGTCTTCTAAATGCGTATATTATACGTATAA**ATG**ATGGGACGAGTCCGACACAATACGTTCTCTATAATTAGAGTATGTAGATCAATTTTCTACCATGACAACAACATAGGAAAGTGATTATAGTATGTAGATCAATTTTCTACCATGACAACAACATAGGAAAGTGATAGCCAACACTAATCCATTATTGTATATCTAAATACTTCCGTCCTTTGTTTTCATTCTGCAAGTTAATAAGACGATATGTTTAGATGCACAATGGCACAAGTGTAGATTAGCACGTGAATCAATATAATTACAATCCATATAATTAGTGGCACAGTGAGTAATTCAAAATCGAAGGCACGAAAAAAGTCAAAAGATATTCAATATTAAAAATTTATAATGCAAAGAAACATGGTTTAATATTCATTGTCATACATTCCAAACTAAGAAAAAATTACAAGTGCATTCGAGTCTTCATTTTTTTTGGGAAATGATCAATAATATTATCATCGGAAACTTGAAAAATACATCTTTCTCAAGAAAAAACAATCATTCAAAAATTGATCACCCATGTTAATTCAGCAATTTACTTTTTAGGTAAGTCATTCTCGAAAACACTTTTTCTACACTTTTAGTATAAATTGGAAGAATCAACATCAACTTGATGAGCAAAATTACAAGTGGATAAGCTTCATTCCTATTTGTTTCAACAAACTTCATTGAAAGGGAATAAAGATTATTGATATCTTG*gaattc*ACCACTACCATCTCGCATATCTTCCATGAATACAGGTTCCCATTGACGTCGAGTTTCTTGTCACACTGGATAATACAGTGTGTGTGGAATCCTCCTTCAGGTTGTTCTTCAGTAGGCTGATGATGTTCTAGTGATACTGCAAGGAATAGAATTACATATGATTCTAATAAGGTCTTCAGTTTCTCGAATATATAATCTTTAAAATCCCCCCCTATTTGAGAATATGTTAGAAAAGCATTTTTTTTTTGAAAACGTAAATAACCGGGTTGGTTAGGGGTAGGTCTGTTTCTGTTTGGAACACGTGG**CAT**GTTTGTTTTTGTTTCCCTCTTCCGTACAATGAATGAAAAGAAATTGTATATATGAGAGGAAGAGGAACTTAAGAGGAATGAGGAACTGAAGAGGGAGGAAGAGGGATAAATAGGTAAACGCGGATAAGATT

>MC#2

ccccgcgtttacctttccacgtggcgtatgcagagtgggcggacggttgagtggggaacacgctttactttaatttaaagtaaagtagcactaagtgggacccacactttatttctttaaagacttaattattaagtcttctagatgcatattttatacgtataaaccactcgtagttttgacgtataattgttcaaataaaatactactagtatttctttataacttcccaaatatagtgaaccaaaattctgaagaagaattagtattcaatcaaatttttcctatttcatgtccaaattattagtatatccaattctttacttgatgtaatcaagagagagagaggggatatacctactccagaatactaaatcaagagagatagaggattttttataagaaaagaaaaaaataagctggttcttaatataagaccaacctaataaagatagaggggtatacctactttagagtataattttagtggggtttcgtttcattattaggtagtgtttgggaacatagatttcatttgaaattatggaattccacattcccaaacataccattaatgaattttgaaagttgtgttgttattctctatttcttatattactttttattttacatcatatatagactaaaattaagaaaagtaataggctagtgttagatcttttgcatgttaaagttaatataaaattaaagtagctagagcaaaaaactcatacgtatgaactaaaggtgaaaattatacacaaaataaggttaatgggataaagaggaaaaatattaagcaaattataagtaaattatttattatagaaaggtaatttttatgtatatgttctttttaagtatactcctatctattactgtctataagtaggccatctacgaaaagacctctattacttaagatttccaccattagcaactattacaattcacaacacgtcacgtcgtcatcaaggtgatggtggtgtgaaagaaggggaagtgaggaaagaggagaaaatgaaaaaaaaaccagcattcaccAtataatctttaaaatcgccccctatttgggaatatgttagaaaagcatttttcttttgaaaacgtaaatatccgggttggttaggggtaggtctgtttctgtttggagcacgtgg**cat**gtttgtttttgtttccctcttccgtacaatggatgaaaagaaattgtatatatgagaggaagaggaacttaagaggaatgaggaactgaagagggaggtagagggataaataggtaaacgcggataagatt

>MC#3

CCCCGCGTTTACCTTTCCACGTGGCGTATGCAGAGTGGGCGGACGGTTGAGTGGGGAACACGCTTTACTTTAATTTAAAGTAAAGTAGCACTAAGTGGGACCCACACTTTATTTCTTTAAAGACTTAaTTATTAAGTCTTCTAGATGCATATTTTATTGATTCATATTGCAAAATTTATTATTTTAAATTTTTCTTTGAAAAGTAATTCTGTAATGTTTTTGAAAAATTAACATTTATGTAAGATATATACAAGAAAATAATGGTCAGTGTTTATTTTTAAGACTGTACTAGTCAGAAATAGAAATAACTAAATATAATGGAGGAGGGTGTATAACATAGTGAAGGTGGATAATATTGCATTGATATTTTACAACGATATCTATGTATTACATACTTATTCATTGACATTATAAGTAACAGATTAGATAATATGTAGGTTTTCGAAGTGGTTAGATTATATGTAATGCAGGAAATATATTAGATATATGGTGGTGCTACTAAGTAATATTTATATGCTTTTTAAACAATTTTGACACATATCATTACGATACCGGGGTCTTCAAGGAGCAAGTCATAACTTTTCTAACTTATTGAGTTATTCAACTTGACTTGTAAAAGTTGTAACACTTCCAACGATATTTGAATTAGCTGAGCATCGCCTCTGAGGTGTAAAAGATAATGTTCGGATAGAAGAAGCAACATTTGTACATATTAATTATAGTGAGAAAAATTCATCCAAGATTCAAGCCCTTTTTTTTTGGGCACATTTTCAATTCATTTCAGTTTAACTCACTTCAACTGATTTCTTCTCACAATTTCAGTTCAGTTCAATTCAGCTCAATTCAATTTAGTTTAGTTTAGTTCAATTCATTTCAATTTAACTCATCCAAAAAGAACATACCTTTATCTTATTTTTCGATATAACTACTCTCAAAATAAGAAAAAACAACAAAATAAACTAAAAGGAAACAAATAGTAGCAAATACAGGACCTGTTGATACACCTGCAAAACTAGACTTGAGTATTACCAAAAATGCACCCCTTTCACTAAGAAGTAAGAACCCTCCATGAGCGCTTTGAATATATAGTTATATACGGAATACTACACTACTTCTCCACTAAAGCTTTCTTTTAATTTCACCTCTTTCCTTTACACTGACATCTGCAAAATTAGAGAGTTCTCTCTGATATTATAGTAAATGGTAAATAAATGTGAACTCCTCCTTGGTGtTGGAtGAATCAAgaattcGTCTCcATTCCaCGTTGCTGTTGCCaATACTGTTTGGAGAGCAAAAAGCATTTTTCTTTTGAAAACGTAAATATCCGGGTTGGTTAGGGGTAGGTCTGTTTCTGTTTGGAGCACGTGGCATGTTTGTTTTTGTTTCCCTCTTCCGTACAATGGATGAAAAGAAATTGTATATATGAGAGGAAGAGGAACTTAAGAGGAATGAGGAACTGAAGAGGGAGGTAGAGGGATAAATAGGTAAACGCGGATAAGATT

>MC#4

CcccgcgtttacctttccacgtggcgtatgctgagtgggcggacggttgagtggggaacacgctttactttaatttaaagtaaagtagcactaagtgggccccacattttatttctttaaagacttactttttaatacaaatatgtatgctactttgtttggtaattgtagggcttatttctaCtaatagtaacaaaattggaaataaattgaattaaatttaatttagttgtagtggagtgaatttaatttagctaaactaaatttaatgtttaactgaattaaatgtaattataagtatactaagatgaactgaaattaagtctaaaagaacatcgcctacatttagctttatactatggatatctaagaataatggcacaatactaaattcatgaagtaccaatattgttatacttaacatagtttgaaattcatttactttcaatacaagtttttaattcatgaagtgggagaaacataaattatattatgggacaaaggaattatagtgagtctttttactttcacgcttgttatttttgggacaaagggagtgtagtgagtttttttactcacacttgctgctggaaaacgttaacctttaatttatttaatcatgaataaaaaaaatagttggtagtcccgaaaaatatatatcgagacaaatctaataagatcacacgtgaatatattttttttaatcatgtatgaggcatttttagtctatccagtggcattgtgattaaattgtcaaaaaaaactaattactcttttattcccatattaatcacaataaatcacttcgaccataatttgtaactaatacataaagaaatattgcattagtgttatccgttgcaaagaaaaatgtatctcgtgttgcaatatagtcaaccgttgcattagaacgttgtatttgcttcaaattgcagtaataatacacttatttgtctaatatgtgtgcttatgtatattttgtgacaccaaatttttatcgttgttactaacagattattagaaatattaataaagaaatttatgcagataaatacgtcagactaataaggtcttcagtttctcgaatatataatctttaaaatccccccctatttgagaatatgttagaaaagcattttttttttgaaaacgtaaataaccgggttggttaggggtaggtctgtttctgtttggaacacgtgg**cat**gtttgtttttgtttccctcttccgtacaatgaatgaaaagaaattgtatatatgagaggaagaggaacttaagaggaatgaggaactgaagagggaggaagagggataaataggtaaacgcggataagatt

>MC#5

CCCCGCGTTTACCTTTCCACGTGGCGTATGCTGAGTGGGCGGACGGTTGAGTGGGGAACACGCTTTACTTTAATTTAAAGTAAAGTAGCACTAAGTGGGCCCCACATTTTATTTCTTTAAAGACTTACTTTTTAAGTCTTCTAAATGCGTATATTATACGTATAA**ATG**ATGGTCTGTATTCCCGACTGGTTATTTCTACTCTTCGTATTTAGTACGATTCTTCAGTCGGGAATTAATTTCT**ATG**GTACCTTTCAGAGTGAGCGAATTATATATATATTTCTTTACCATTATAAAGTATATGTTGCAACAAATGTAAGAAGTTTATCCAAAAAAAAAAAAAAAAAAAAATGTAAGAAGGTGTACCAAATTTTTGATATAAGTAGAGGTATATAGTGAAGTAGTTGTTCAAATAGGGGATGTAAATAGTGGAAAATATCTAACAAGAAAAAGTATTCCTTCAAATTTTTAAAGATCTTCCTATATGTAGTAGCCAATAGAGATACCAAAAATTCAAGATAAAGAGAAGACAAAGTGTAAAAGTGAGTCATTTTTGTTTACCTAGGCAAAAAATAATTATGTGCATCTAATAAGTGAGATAAGATAGTACAAAATTAAATAAATATAATAAATAAGGAATAAAGTAAATATAAGAAAATCTTTTGAATATAAATTTAAACAGAAAGTAGGAGAATCtTTTTAAAAAATAAGGAAAATTAAATATATATTTTTAATTGAAAAATATCTTAACGAGTTGGGGAAGTAAAAGAACTCTTAACATATCAAAAGTAGAAATTGATTCTAAGATCTCTTAAAACCAAGATATATGCTAAACTATTAACTTAACCTTCACCCTTTAGAAAGTAGCAAATATGGAGGGTGTGAAAAAATCAAAACTCCAATATGATGCAATTATGAGTATCTTGATTTGTGGGTACAATAGGAGAGGATAAAAAATAAAGCACATGACCCATTCACTTACACACATATTTCGTGGAAGTACACTTCACCAATCTAGTCTCTATCTAGATACTCCCAAGTCCCAACTTAAAGAATATTTTGTTTTTACTTCCTCCATTTTTTACTACTTTTTCCGTTTCATAATTAGGGATATAAGTGGGGCGGGTTAAGTAAGAGACTTGTCCCATCTCCGTCAAATTGGGGAGGGGATGGGTCTAGATTTGGCAGGGATGGGGATTAAAATTGTACCCGCAATAATGGGTGGTGAAGCGGGTGTGAATTTTCAGTTTCTCGAATATATAATCTTTAAAATCCCCCCCTATTTGAGAATATGTTAGAAAAGCATTTTTTTTTTGAAAACGTAAATAACCGGGTTGGTTAGGGGTAGGTCTGTTTCTGTTTGGAACACGTGG**CAT**GTTTGTTTTTGTTTCCCTCTTCCGTACAATGAATGAAAAGAAATTGTATATATGAGAGGAAGAGGAACTTAAGAGGAATGAGGAACTGAAGAGGGAGGAAGAGGGATAAATAGGTAAACGCGGATAAGATT

>MC#6 CCCCGCGTTTACCTTTCCACGTGGCGTATGCTGAGTGGGCGGACGGTTGAGTGGGGAACACGCTTTACTTTAATTTAAAGTAAAGTAGCACTAAGTGGGCCCCACATTTTATTTCTTTAAAGACTTACTTTTTAAGTCTTCTAAATGCGTATATTATACGTATAA**ATG**ATGGTCTGTATTCCCGACTGAAATAGCTAACCACCCTTCTTTTCCTTTTTTATTCTTAAAAGAATGAAACACTTCATGAGCTAATAGAATATTATCTTGAATTTGTCTTTCAGGGACAAAAGCCTCTTGTAAAGGATGAATAATCCGACCCAACACTACTTTCAATCTATTAGCATAATTTTTGAAATAATTTCATAAATAGTTGAGCAAAGACTAATAGGTCGAAAATGATTTGGGTTAGAAGGAGCTTCTATTTTAGGAATTAGAGTGATAAAAGTATGATCGATTAATTTCTTTTAAAATTTTTCCTGAGTGAAAAAAGGCTTTAATAGCCCTATACACTGAGTTTCCCACAAGAGTCCAATACTTTTGATAGAATAAAGGAGTAAAACCATCTGGACCCGGAGATTTATCCGGTGCTAGATCAAACAGAGCAGATTTGACTTCTTCAATCGATATATCAGCGCATAGGTCTATGTTATCATTTTCAGAAATTATACACTCTAATAAGGATAGGTCAGAGTCATAATTAAAACCACATTCCATATTCTTAGCAAATCTGTTCTAAAACGCATTAGTAATACATTAtTCTACTAATTTTGGTTGAATGACTAAACGATCTGACGGATCTTTAATACAAGTGATTAAATTACGATTTCGTCTAATCGAAGCACACGCATGATAATACTTTGTGTTTGCGTCCCCAAATTTTAAATTATTAGACTTAGCCTTGATTTTCCAATAGGAGTCTTGAAAGTCTAACAATTTTGTTTGCTTAGCCAAAAACAAATTTTGTTGTCTAATTAGATGAGGGCAATCATTATGTTCAATTAATTGATTTTGAATTCTAACCAACTTTCCTTCGACACTACTTAATTGTCTAGCTATATTGCCAAATCTGGTAGTGACCCAAAATTTAGCCTTTTCCTTTAAAAGTTTGCACTTTTTAATTAAGCAATACATGTTCGAACCTTGAAATCTTTGACACCAAGTTTTCTTGATTAACACATCAAAATCCTTTCGAGTCACACTGGATAATACAGTGTGTGTGGAATCCTCCTTCAGGTTGTTCTTCAGTAGGCTGATGATGTTCTAGTGATACTGCAAGGAATAGAATTACATATGATTCTAATAAGGTCTTCAGTTTCTCGAATATATAATCTTTAAAATCCCCCCCTATTTGAGAATATGTTAGAAAAGCATTTTTTTTTTGAAAACGTAAATAACCGGGTTGGTTAGGGGTAGGTCTGTTTCTGTTTGGAACACGTGG**CAT**GTTTGTTTTTGTTTCCCTCTTCCGTACAATGAATGAAAAGAAATTGTATATATGAGAGGAAGAGGAACTTAAGAGGAATGAGGAACTGAAGAGGGAGGAAGAGGGATAAATAGGTAAACGCGGATAAGATT

>MC#7

CCCCGCGTTTACCTTTCCACGTGGCGTATGCTGAGTGGGCGGACGGTTGAGTGGGGAACACGCTTTACTTTAATTTAAAGTAAAGTAGCACTAAGTGGGCCCCACATTTTATTTCTTTAAAGACTTACTTTTTAAGTCTTCTAAATGCGTATATTATACGTATAA**ATG**ATGGTCTGTATTCCCGACTGGTTATTTCTACTCTTCGTATTTAGTACGATTCTTCAGTCGGGAATTAATTTCT**ATG**GTACCTTTCAGAGTGAGCGAATTTCCGCGAAGCTATCCAGCCTTGCTAGCCGTTTCGACGAGCTGTTTCTTGCGTTACAACAAACGGAACCCTCCCCACTTTTTTTTCCTTTTCTGAATTTATGCCCACAACAATGTACTCCCTCCATTCCAGAATAGTTTGAAAACTTTTTTTTTGTCCCAAATTAGTTTGAGCATTTTCATTTTTTTGATATGGACCCACTATTACATTAAGTTAGTCTTAAAAGGTCAGCCTACTTTTATTTCACAATTATGCACGATGTAAGTCTAGTATAAAGAAGGGAACATGCTACCTTTCTAAATAAAATGACTATTTTAATCTATTAAGTGACAAATCCATTCAATAATTATATTATTTAATCTATTTAGTGACTAGTTAGTTAAATCCATTCGATAAGACTGTTTGATTATTTGTGATATGTAGTTCGAGATTCTAATGTATACAAAGAAAAGAGAGAAGAAGGAATGACTAAGTTTGTGTGAGATATTTAGGGATAATGTTAGGTTAGAGTGAGATGGAATGGGATTGTTAGTAAAATGTAGGCTAATGTTTGTCTAATTTTATATTAATATTAGACAATCTGTCCTAATAATTTGTCTATTAGATTATAGTTTTCTAAAACTCTTTTGGCCCACACTTAATCTCTATTATTAATATAAAAAAACAATTAACTTTCTTTCTTTTAAAAAAATATACTCAACTAACTTTTGTCACATGTACTCCCTCATTTTTTTTCCATTTATGTTTGTCCCAAATTTAATAGATTTTTCATTTTCTTATTTGGTATGGATCCCCCAATTAGAATATACTTTAGCTTTCATTTTTATTATATCACCCACTTCCTCACATTCTCTCTCCTCAATAAGCAATTCATGAATAGTGTGTTCTTCAGTAGGCTGATGATGTTCTAGTGATACTGCAAGGAATAGAATTACATATGATTCTAATAAGGTCTTCAGTTTCTCGAATATATAATCTTTAAAATCCCCCCCTATTTGAGAATATGTTAGAAAAGCATTTTTTTTTTGAAAACGTAAATAACCGGGTTGGTTAGGGGTAGGTCTGTTTCTGTTTGGAACACGTGG**CAT**GTTTGTTTTTGTTTCCCTCTTCCGTACAATGAATGAAAAGAAATTGTATATATGAGAGGAAGAGGAACTTAAGAGGAATGAGGAACTGAAGAGGGAGGAAGAGGGATAAATAGGTAAACGCGGATAAGATT
