# Supplementary material for: Virus-mediated export of chromosomal DNA in plants
Source: Nat Commun. 2018 Dec 13;9:5308. doi: 10.1038/s41467-018-07775-w (PMC6293997; doi:10.1038/s41467-018-07775-w)
Supplement: Supplementary file 6 — Supplementary Data 3 [file 41467_2018_7775_MOESM6_ESM.docx]

**Supplementary Data 4**

Fasta sequence of scaffolds assembled from aligned reads sequenced from a BCTIV-infected *Beta vulgaris* plant.

>scaffold_1

ACATATTCTCAAATAGGGGGGGATTTTAAAGATTATATATTCGAGAAACTGAAGACCTTATTAGAATCATATGTGTATATATATATATATATATATACATATATATATATATATACATATATATATATATACATATACATATATATATATACATATACATATATATATATACATATACATATATATATATATATATATATATACATATTCGGAAAATATACATTCAGACGAATCTAACAAGATCCCACATACATATGTTTTTCCTTAACTATAAATCACCAAACATAAACTAAGTAAAGTTGGTGAATAGTGTAAAAGTCAAACCGTTGCAAGTAAAAAGAAACGGAGGAAGTATAACCCAAGACAAACAAATGTCATGATGTCACATGTGACATGTATTCTCCCCTCGTCACATAATCAAATGAGACATTAAACAATAAAAATATGTGTTCCAGTAATCCAACCCATGAGATCTCATTTGATATGCTAATGCACGAACCAACTACATACAATACTCATGTTGGTCTTTTTGAGGGAAAAATCATTTAAAATAAATACAATATTTCTATTTTTGATGCCTTCAAATACACGTTGATAATAATCTAGGAATATTGTCCGGGATAAGAAATGGTTTATTTATAAATCTAACAAAAAAAAAAGAAAATTTGAATTTTGAAATATTTTCCTTTTCTAACAATGACTAAAGGAGATGATCTAATTTTAATATAAAAGTAGACCTAGTTGCTAAATGGATTAAATTAGTCCTTGAAAAAAATTAACCTAGTTACTATTGGGCTATTTTTCTTTTTAATATTGCATTTGCCATTGCCTAATTTTAATTTAAAGGTGAACGATTTCTTATAAGACAAACTCTTCCTTTTAAGCCCCACCATCGACATCTCTACATCCTTTTGTTTATAGGAATTTCCCTATGAATTAGTTTCTGATAATAATTGTTAACAGCTAGTAATTATTAACAACTCTGTCAGAATCTATTTTGGACATGCAACTATTGTCAGAATCTTGAAACATAAAAAATAGTAATGTAACAGTGTTAGATTAAGGAAAATCCAATACCGTCGTTTTGGTTTCAAATGAAAAAGTAGATATCATGTTAAATACATAGAAATATACTATAAGCATTACTATTGTATGATTCAAAATCAAAACTAAAATAGAAATATATCCATTCAAAATGTGGGGCCCACTTAGTGCTACTTTACTTTAAATTAAAGTAAAGCGTGTTCCCCACTCAACCGTCCGCCC

>scaffold_2

ACATTTTATTTCTTTAAAGACTTACTTTTTAAGTCTTCTAAATGCGTATATTATACGTATAAATGATGGTCTGTCCCCCTAGTTGTGAATGTGTCAAGATTTCAGATAAACAAACTAAAAAAATCAGAAGAAGTGCTAATTATAATATCAATAACAATAATAATATAATATACTCCTATTAAAATTACACTTTTAATTATAATTATAATATCAATAATAATAGGTTTAATAATAGTGTTTATCAAAATCAACTTGAAATTTATTTATGTTAGTAGTACTTGCAAACTCTTAATGTAGCTTACACCCTTCTTAATCTAACACTGTATAATAAATTATAGTTTTATAAATTTCTAATAAAGAAATAATAAATATCAAATAGGATCTAGATATCCGAATTTACAAAATTGGATATCCAATTTGGTGTTTATGGGTTGGATATCTAAATATTCAAATAACTAAAGTGGATTCCAAATATTCACTTTTTCGGTTCGTTTTGTTCAACCTTATACAAAAGTAAAAGCATCGTGAATTACAACTTTTGTCATACATAGGAGTGTACCTTATAACACCTAATGTTTTCTTTTGTTCATTGTTCGTTTAAACCCTCAAAATGTGCAAAATTTGAATAATAGGAGGAGTTTCAAATGAGCAAAAGATAGCCTTAACTTCTCATGTGTATATGACATACGTTACACCATAGACTCTACTCCCTAGCTAGTGTACATTGGATATACTCTGGAGAAGAGGGGATGAGTACTGGAGGGGAGAGATAATATTAATTAGATTTGATAGTAATTAAATATATTTCCTCAAAAGAAAAGAAAAAAAAATAGAATAACAGCACTTGGAATACTTAATTTAAATTACGCGCACAAAATAGGATGCAAATACCAAAAGGAAATCTATATAAATGGACAATGCTACATCTATCCCGTTAATAATTAATCTACTAAAAATAAAAAATTAGAAAACTATTAAAATTAATAAATTATACTCCCTCTGTTTTTTTTTATCTTTCTCACTTAGAATCTTGACACTATTCATGTATAGAGAGAATGTTTCTGTTTGGAACACGTGGCATGTTTGTTTTTGTTTCCCTCTTCCGTACAATGAATGAAAAGAAATTGTAT

>scaffold_4

TGCTTTTCTAACATATTCTCAAATAGGGGGGGATTTTAAAGATTATATATTCGAGAAACTGAAGACCTTATTAGTCTGACGTATTTATCTGCATAAATTTCTTTATTAATATTTCTAATAATCTGTTAGTAACAACGATAAAAATTTGGTGTCACAAAATATACATAAGCACACATATTAGACAAATAAGTGTATTATTACTGCAATTTGAAGCAAATACAACGTTCTAATGCAACGGTTGACTATATTGCAACACGAGATACATTTTTCTTTGCAACGGATAACACTAATGCAATATTTCTTTATGTATTAGTTACAAATTATGGTCGAAGTGATTTATTGTGATTAATATGGGAATAAAAGAGTAATTAGTTTTTTTTGACAATTTAATCACAATGCCACTGGATAGACTAAAAATGCCTCATACATGATTAAAAAAAATATATTCACGTGTGATCTTATTAGATTTGTCTCGATATATATTTTTCGGGACTACCAACTATTTTTTTTATTCATGATTAAATAAATTAAAGGTTAAAGTTTTACAGCAGCAAGTGTGAGTAAAAAAACTCACTACACTCCCTTTGTCCCAAAAATAACAAGCGTGAAAGTAAAAAGACTCACTATAATTCCTTTGTCCCATAATATAATTTATGTTTCTCCCACTTCATGAATTAAAAACTTGTATTGAAAGTAAATGAATTTCAAACTATGTTAAGTATAACAATATTGGTACTTCATGAATTTAGTATTGTGCCATTATTCTTAGATATCCATAGTATAAAGCTAAATGTAGGCGATGTTCTTTTAGACTTAATTTCAGTTCATCTTAGTATACTTATAATTACATTTAATTCAGTTAAACATTAAATTTAGTTTAGCTAAATTAAATTCACTCCACTACAACTAAATTAAATTTAATTCAATTTATTTCCAATTTTGTTACTATTAGTAGAAATAAGCCCTACAATTACCAAACAAAGTAGCATACATATTTGTATTAAAAAGTAAGTCTTTAAAGAAATAAAATGTGGGGCCCACTTAGTGCTACTTTACTTTAAATTAAAGTAAAGC

>scaffold_5

CCCAAGAATCGACGGACTCAACGCCCCAAAGCGAGCATTCGAGTACATCACCAAGGAGGACACGAGCCCTAGAATTTTCCTTTGAATCTCGTACTACAACGGACAATTCTATAAACTAAACTGACAATCTTATCGAATGAATTAAACTAGTCACTAAGCGGGTTAAACTAGTCACTAAATGAATTAAAGAATGTGGGAACTAAATAAATTAAACTAGTCACTAAAAAAAATTAAGCTGTCCACTAAATAGATTAAAGTAGTCATTATATTTGGAAAGACGGTATGTTCTCTTCTTAATATTGAGTTTGGATCGTTGTCTAATAATTTTAAACTAAAAGTAGGTGGTCTCTTACAATACCAACTGTAGGACATTTGGGTAGCTAGATTGGTTTATGCTACAGAAAAAAAAAGTCTTGCAATGAAGGACACGATGAATTTTCTTCACCTGAATTTCACTTAATTAATTATATAGAACTATAAGCTTATACTAAGTATTAGCTTATCTAGACATATATTAACTTACTTGAACTTAGTAGACTTAATTTTCGCCTATATTTGAATATATTTACCCTTGTCAAGCCTTATTAGAACTTATGATAGATCTTTATTAGCATTCTTGGAAAGTGATTCTTACTTCATAGTTCATATATATGTTTCCAAATAAGTGCAACAATTATTTGACTTTCACGTTTGTTGACGCACAATTTTGACCATTAATATCATCAATTATATATTAGAAGGTTGATATTATGAAAATACTCAATAATGTGTATATTGTTGTGTGCTAGGTCACTATCCATAAAACTTCGTATTATTTTTTTAAATTTCGATAAAATTATATTATGGTCAAAGTAAAGTATATTAATAAGGTAAACACTGATTAGAAAACACAGAAACGGCCAAAAAGCGCGGAAATAACCAGTCGGGAATACAGACCATCATTTATACGTATAATATACGCATTTAGAAGACTTAAAAAGTAAGT

>scaffold_7

TCTCAAATAGGGGGGGATTTTAAAGATTATATATTCGAGAAACTGAAGACCTTATTAGAATCATATGTAATTCTCCCCTTCTTTGTACTTTGTACTGGTGTCGCAATTGTATCATAATCCTTTCCGACTATATTACACTTATGTTTTCTTTTGCCTATTCCACTCTAATTTTTGTCTATTCGCACATCAAATTTAATCATGTCTTTTCTAGTATACAAATATAAATGGTATTAATCCAAATATTCTATAGTTGAAATGATTTTACAATGGCTACAACAAAATGATAGCCATGATAACTTGTCTAATTTAGACCATTCATTTTAATATAAAATTTACACCAATTATCTAATTAATTTTTTACAATTAAACTAAAACTAAATCTAGAAGTTATATATTAATAAGTTCGCAATTAACAATTCACCAACTTTTCGACAATTTTTTTTAAATATTTTAAAGGTCAATGAAATCAATGGATAAGACGAGCTGTTACAAAAAAGAGAGTTGAAATTAAGTAATTTTAGTGACTACAATTTTAATATAGCCACTAAAAAAACTTACTATATAGTTGTATATTCTTCATTTCTTCATATATCAACTTCTACTAATAGCTAGTTAATGATTTTAATAATCCAAGTTATATATTGACGAATATGTCAAGTTTAAAGGTAAGAAAAATAATAGAGAGATAATTTTTTTAATGCTTTTTAATTTTTCTTGTTCTTTCACCCAAAAAAAAAATGTTTGTTCTTCTAGGAGACCATCCACATCAGTTTGGTTCGTTTCCTGTTCTTTAGTGGCCAATTAGTTAATTGGTGATAACTGGGCTTAACCTTTGCGGCTTTTCGGGACTTGTTGTAACGCAAGAAACAGCTCGTCGAAACGGCTAGCAAGGCTGGATAGCTTCGCGGAAATTCGCTCACT

>scaffold_33

TGGAGACGAATTCTTGATTCATCCAACACCAAGGAGGAGTTCTTCAGTAACATACGAGAGTCCTGCCCAACAGACTTGAACAGTATATGATGATCAATCACACATAAATATGGTCATATGAGATCTTATTTAATACGTCTCAGTGTGTAATTTTTTAGTATTTCATTTGCATAATTTTTACAAATACAAGTACATAACTAAAGTTATTTATGGGCAAAATTGTGCATTGACATTAGTATGAAAGAATTCAACGACAATATATTGGGAACTTGGGATAATATGGGCTTCTTGAAAAATTTGTTCCAAAAACATGCTCAGCATCTATGTGACTATGTTATTTTTTAAGGGATCTATGACTAAAATTTTTGTAGGATTTAACATTCTTCCGCCTGATTTTTTTTTTAATTATTATTCTTATATCTACTTTTTCTCTCACAATAATATTTCGTAACTCTCTGAAAAGGTAATACTATGGTGACAATAACAAAAATATAAACTCAATTTCAGAATTTGTGAACTAATAAAATCAAATCATTATATACTTCACATCATAATGTGGGGCCCACTTAGTGCTACTTTA

>scaffold_56

AGTTGAATCAAACAAAAATTTCCTTCATAAAAACTACGATCGTCAAATAAATCTTGCAATGTGGAACATCTCCCGCTTTTATAGGGATTGAAAATAAGTTAACATTATAGGACCACATTACTAGATAAAGTACAAATTCATCGATATTAGTAATTTCAAAAATTATTAAAAAAAAGACGGTCTAATTTTTGTATAAAATTAGACATAATCACTAAATGGATTAAACTAGTCACCGGGCCCACTTAGTGCTACTTTACTTTAAATTAAAGTAAAGCGTGTTCCCCACTCAACCGTCCGCCCACTCAGCA

>scaffold_59

GCGCATTATACATGGTTCAAAAGGCTAGTATAATTATAAATATATATAGATTATATGCGTGTTAAAATTGTACATAGGAATGCTTAAGATTAGAGTTGTATATGGCCCGATCTAGCACAAGTTTGGTCCGACATGTCATAACCGAGACTAGCTGAACACGGCAAGTTGGGTTGTGACATATGGAGTGTGCCTAGGCCTCCTTTTTTTAAAATTCAGCATAATCATCCTCCTTCAGGTTGTTCTTCAGTAGGCTGATGATGTTCTAGTGATACTGCAAGGAATAGAATTACATATGAT
