# Supplementary material for: Virus-mediated export of chromosomal DNA in plants
Source: Nat Commun. 2018 Dec 13;9:5308. doi: 10.1038/s41467-018-07775-w (PMC6293997; doi:10.1038/s41467-018-07775-w)
Supplement: Supplementary file 8 — Supplementary Data 5 [file 41467_2018_7775_MOESM8_ESM.pdf]

Figure 1

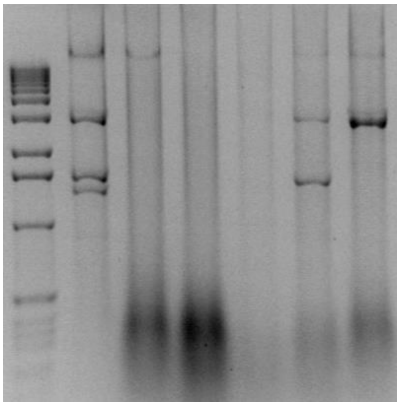

Figure 2

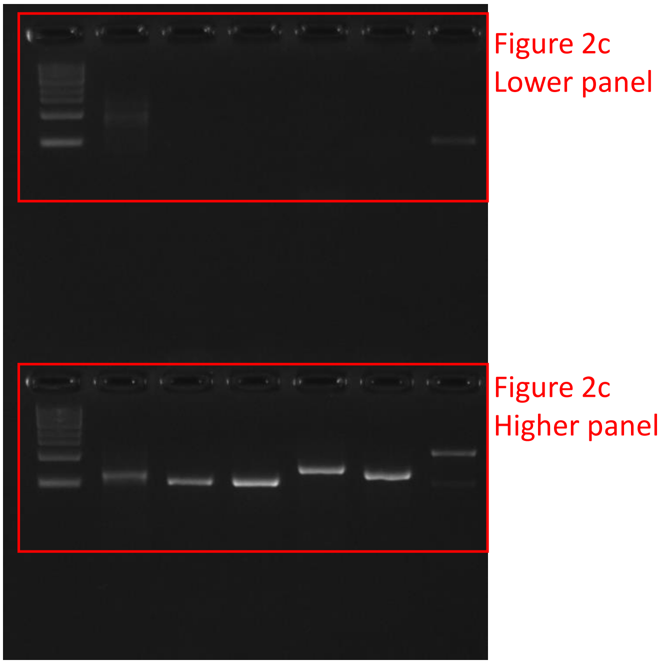

Supplementary Figure 3b

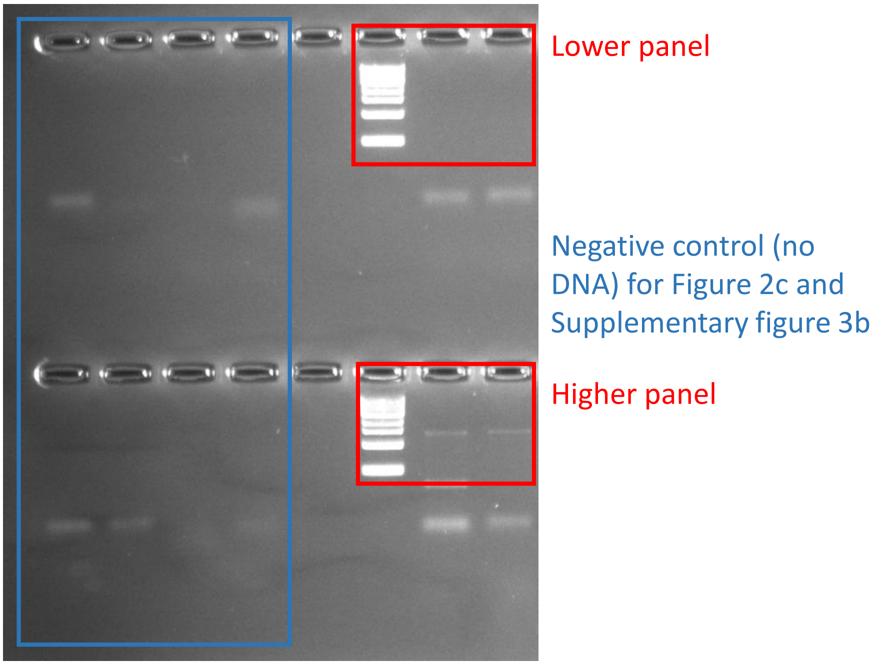

Figure 2f source data

	Means		s.d.	
	healthy	BCTIV	healthy	BCTIV
#1	1.13	6.86	0.73	0.89
#2	1.51	4.41	0.67	0.21
#4	2.01	13.32	0.24	0.07
#5	1.16	2.86	0.80	2.10
#7	1.12	3.77	0.60	0.51
#33	2.03	4.30	0.11	0.23
#56/59	1.01	4.85	0.36	0.46
C1	nd	14.20	nd	0.31

Figure 3

Southern blots probed with virus-specific (red) or minicircle-specific (yellow) probes

Figure 3a.
Beta vulgaris

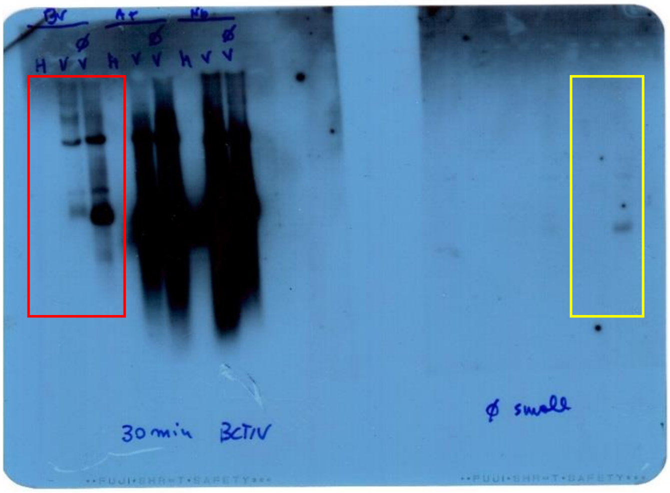

Figure 3b
Arabidopsis

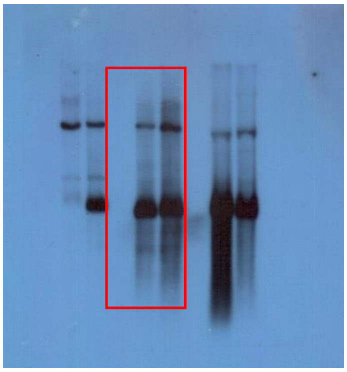

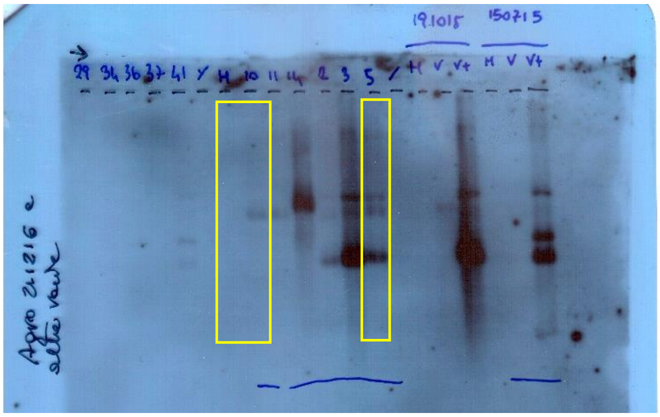

Figure 3c
N. benthamiana

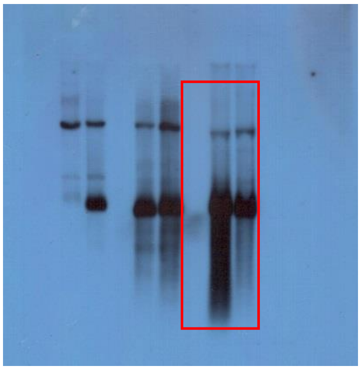

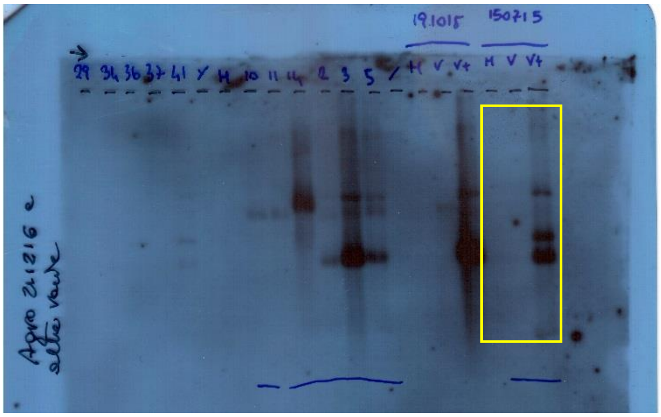

Figure 3d

Southern blot: purified virions and N. benthamiana plants used for virion purification with virus-specific (red) or minicircle-specific (yellow) probes

BCTIV probe

Minicircle probe

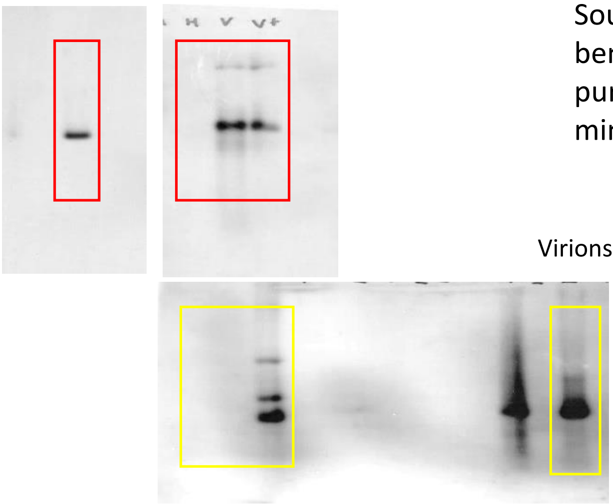

Figure 3e

RT-PCR from beta and benthamiana

Host transcript

25S

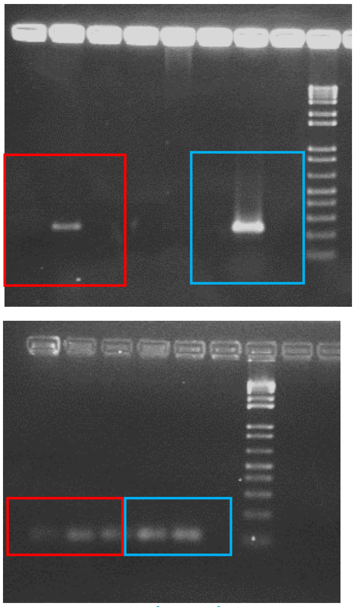

Beta N. benthamiana

Supplementary Figure 1

Panel 1a

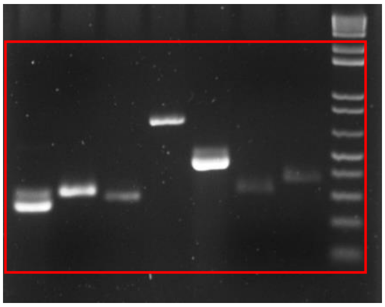

Panel 1b

B. vulgaris

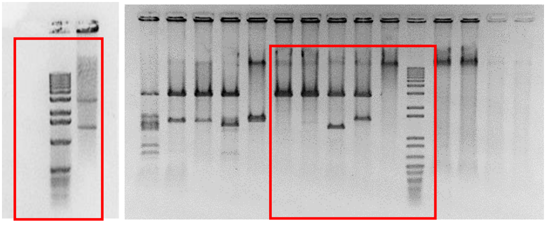

Arabidopsis

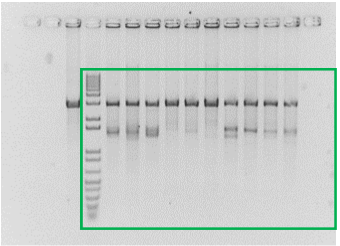

N. benthamiana

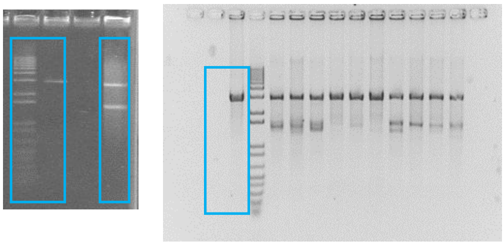

Supplementary Figure 4

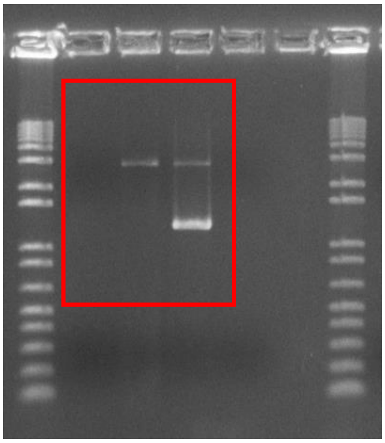

Panel 4a
PCR from plants inoculated with BCTIV and with BCTIV + minicircle

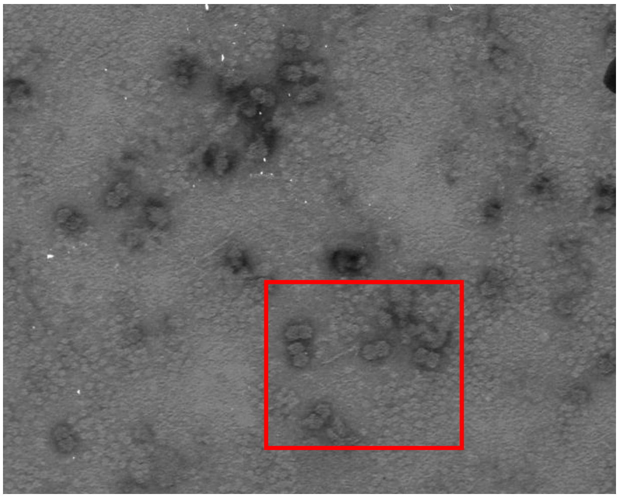

Panel 4b
EM purified virions

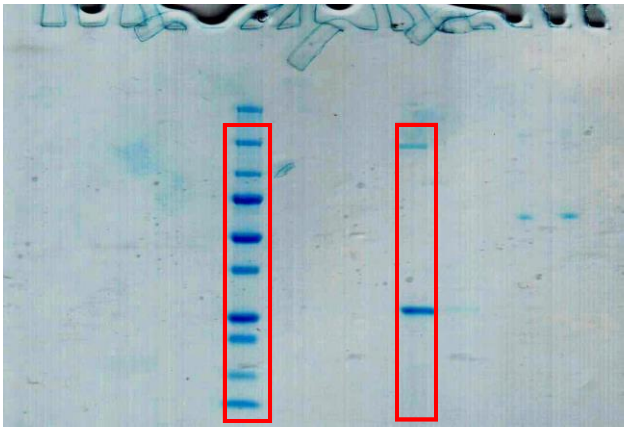

Panel 4c
SDS-PAGE of purified virions
